# Genetically Regulated Bilirubin and Risk of Non-alcoholic Fatty Liver Disease: A Mendelian Randomization Study

**DOI:** 10.3389/fgene.2018.00662

**Published:** 2018-12-18

**Authors:** Lei Luo, Ping An, Xinyong Jia, Xiaobian Yue, Sujun Zheng, Shuang Liu, Yu Chen, Wei An, Cheryl A. Winkler, Zhongping Duan

**Affiliations:** ^1^The Department of Infectious Diseases, The First Hospital of Lanzhou University, Lanzhou University, Lanzhou, China; ^2^Artificial Liver Center, Beijing YouAn Hospital, Capital Medical University, Beijing, China; ^3^Basic Research Laboratory, Center for Cancer Research, National Cancer Institute, Basic Science Program, Leidos Biomedical Research, Inc., Frederick National Laboratory for Cancer Research, Frederick, MD, United States; ^4^The Department of Medical Laboratory, The Second Affiliated Hospital of Luohe Medical College, Luohe, China; ^5^The Department of Intensive Care Unit, The Second Affiliated Hospital of Luohe Medical College, Luohe, China; ^6^Beijing Municipal Key Laboratory of Liver Failure and Artificial Liver Treatment Research, Beijing, China; ^7^The Translational Hepatology Institute and College of Basic Medicine, Capital Medical University, Beijing, China; ^8^Department of Cell Biology and Municipal Laboratory of Liver Protection and Regulation of Regeneration, Capital Medical University, Beijing, China

**Keywords:** bilirubin, NAFLD, variant, *UGT1A1*, mendelian randomization

## Abstract

Mildly elevated serum bilirubin levels were reported to be associated with decreased risk of non-alcoholic fatty liver disease (NAFLD). Whether this is a causal relationship remains unclear. We tested the hypothesis that genetically elevated plasma bilirubin levels are causally related to reduce risk of NAFLD. A total of 403 eligible participants were enrolled. NAFLD was determined by liver ultrasonography. The uridine diphosphate glucuronosyltransferase 1A1 (*UGT1A1*) gene variants (UGT1A1^*^6 and UGT1A1^*^28) were genotyped through sequencing. We applied a Mendelian randomization approach to assess the effects of genetically elevated bilirubin levels on NAFLD. NAFLD was diagnosed in 19% of participants in our study (NAFLD = 76; Non-NAFLD = 327). The variants of UGT1A1^*^28 and UGT1A1^*^6 were strongly associated with increased total bilirubin (TB), direct bilirubin (DB), and indirect bilirubin (IB) levels (each *P* < 0.001). These two common variants explain 12.7% (TB), 11.4% (IB), and 10.2% (DB) of the variance in bilirubin levels, respectively. In logistic regression model, after multifactorial adjustment for sex, age, aminotransferase (ALT), white blood count (WBC), and body mass index (BMI), variant UGT1A1^*^28 (OR = 1.39; 95%CI: 0.614–3.170; *P* = 0.43) and UGT1A1^*^6 (OR = 1.64, 95%CI, 0.78–3.44; *P* = 0.19) genotypes were not significantly associated with the risk of NAFLD. Moreover, the plasma bilirubin level (TB, IB, and DB) were not significantly associated with the risk of NAFLD (*P* > 0.30). A Mendelian randomization analysis of the *UGT1A1* variants suggests that bilirubin is unlikely causally related with the risk of NAFLD.

## Introduction

Bilirubin, the by-product of hemoglobin catabolism, is generally considered to be a lipid-soluble waste product that needs to be excreted (Fujiwara et al., 2018; Hamoud et al., 2018). However, bilirubin levels may play an important physiological role as antioxidant (Stocker et al., 1987). It has been reported that elevated plasma levels of bilirubin are associated with reduced risk of non-alcoholic fatty liver disease (NAFLD) in case-control studies (Hjelkrem et al., 2012; Salomone et al., 2013) and in retrospective epidemiological studies(Chang et al., 2012; Tian et al., 2016). However, whether these associations reflect a true biological protective effect of bilirubin rather than confounding or reverse causation remains unknown. Whether bilirubin has negative or positive impact on NAFLD has important implication for clinical management. If positive, that means hyperbilirubinemia has a protective effect on NAFLD and *UGT1A1* gene can be used as a gene target for the treatment of NAFLD. An artificially increasing plasma unconjugated bilirubin strategy as “Iatrogenic Gilbert syndrome” were proposed for reducing cardiovascular disease and cancer risks (Mccarty, 2007; Schwertner and Vítek, 2008).

**Figure 1:**
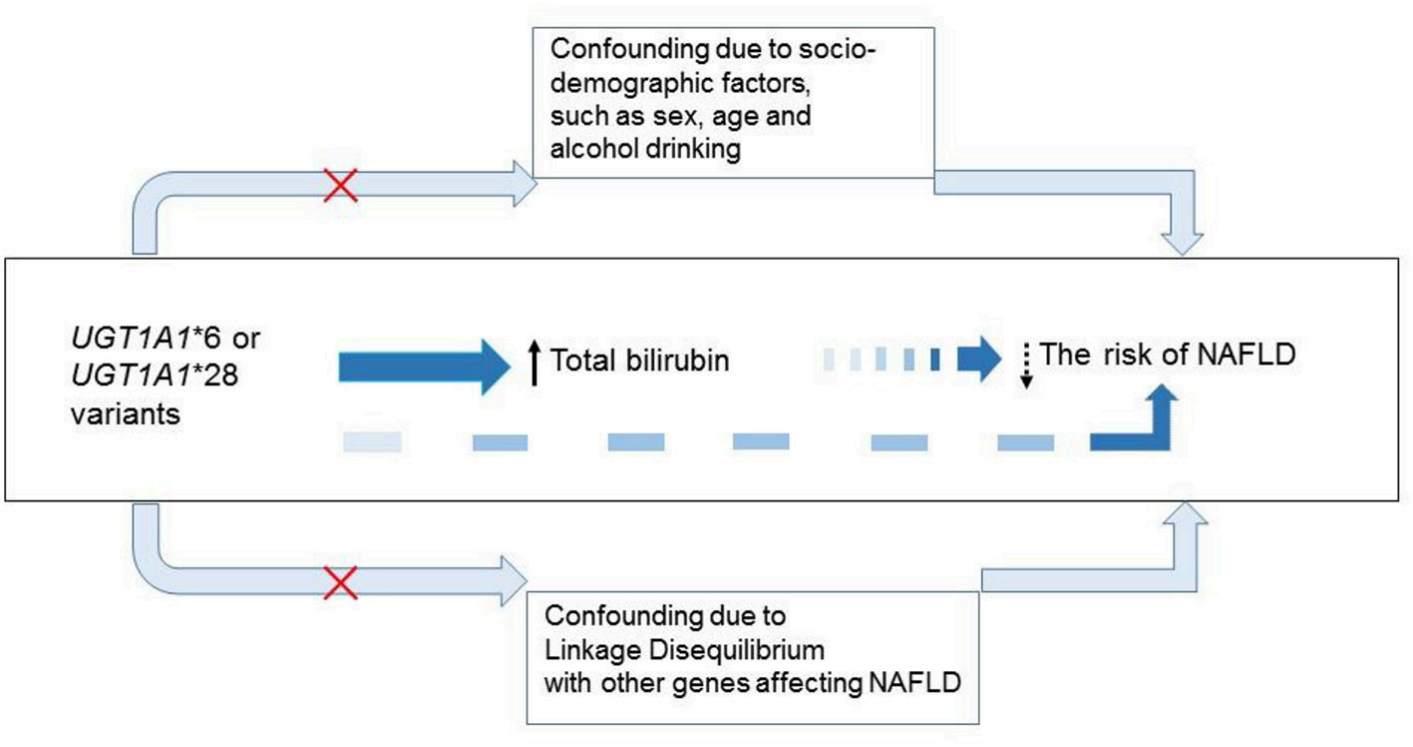
Application of the Mendelian randomization framework in assessing causal role of bilirubin in NAFLD. According to the Mendelian randomization framework, if a genetic variant (*UGT1A1*) reliably governs a disease risk factor (Bilirubin), and there is a causal association between the risk factor (Bilirubin) and disease end-point (NAFLD), then the genetic variant (*UGT1A1*) should itself be associated with the disease end-point (NAFLD). Confounding factors, such as socio-demographic and disease status may affect the total bilirubin levels and the risk of NAFLD by modulate other biological pathways, but do not affect the random distribution of UGT1A1*6 and UGT1A1*28 variants. UGT1A1*6 and UGT1A1*28 were also not in linkage disequilibrium with known genes (e.g., *PNPLA3 and TM6SF2)* that predispose to NAFLD.

Mendelian randomization is an epidemiological method based on the fact that random assortment of genetic variants during meiosis yields a random distribution of genetic variants among individuals in a population (Emdin et al., 2017; Paternoster et al., 2017). The Mendelian randomization approach is ideal to assess the causal relationship of an intermediate phenotype with a disease phenotype by avoiding confounding by reverse causation, a limitation inherent in observational epidemiological studies (Johansen and Hegele, 2013; Ding et al., 2017; Holmes et al., 2017) (Figure 1). Genetic variation in the uridine diphosphate glucuronosyltransferase 1A1 gene (*UGT1A1*) is the major cause of hyperbilirubinemia (Johnson et al., 2009; Fujiwara et al., 2018) and is therefore suitable for exploring whether elevated bilirubin levels is a direct cause of reduced risk of NAFLD using a Mendelian randomization approach.

Herein, we tested the hypothesis that *UGT1A1* variants (UGT1A1^*^28 and UGT1A1^*^6) are associated with higher plasma bilirubin levels and subsequently protect against NAFLD, using a Mendelian randomization approach. We first investigated whether the UGT1A1^*^6 and UGT1A1^*^28 variants were associated with elevated plasma bilirubin [total bilirubin (TB), direct bilirubin (DB), and indirect bilirubin (IB)], and explored the effect of genetic variants on bilirubin levels. We next tested whether elevated baseline plasma bilirubin levels (TB, IB, and DB) were association with decreased risk of NAFLD. Finally, we investigated whether these variants were associated with reduced risk of NAFLD.

## Patients and Methods

### Study Participants

The study population consisted of 446 Han Chinese adults who visited the second affiliated hospital of Luohe Medical College for routine health checkups from June to July in 2017. Among these participants, we excluded 43 participants who met one of the following exclusion criteria; (1) a medical history of CLD [viral hepatitis (*n* = 8), excessive alcohol consumption or cirrhosis]; (2) abnormal liver functions [Alanine aminotransferase (ALT) or Aspartate aminotransferase (AST) >50 U/L (*n* = 3)]; (3) Hemolysis signs [Hemoglobin (HB) < 100 g/L (*n* = 5)]; (4) evidence of infection or biliary obstruction or pregnancy: [White blood count (WBC) >10 × 10^9^/L (*n* = 8); Total bile acid (TBA) >15 umol/L (*n* = 6); Glutamyltransferase (γGT) >80 U/L (*n* = 1), or alkaline phosphatase (ALP) >120 U/L(*n* = 6); pregnancy (*n* = 6)]. The eligible sample size for analyses was 403 (NAFLD = 76; Controls = 327) in the present study.

The study was approved by the Ethics Committee of Beijing YouAn hospital, Capital Medical University and a written informed consent form was obtained from all studied participants.

### Clinical Data

All individuals underwent an abdominal B-type ultrasonography (Siemens S2000, Germany) performed by a trained sonographer. The diagnosis of NAFLD was based on the guidelines developed by the Fatty Liver Disease and Alcoholic Liver Disease Group, Chinese Society of Hepatology (Zeng et al., 2008). Ultrasonographic diagnosis was based on internationally well-accepted Criteria: liver-to-kidney echo contrast, parenchymal brightness, deep beam attenuation, bright vessel walls, obscure hepatic vessel structures, visibility of the diaphragm, and the neck of the gallbladder (Lin Y. C. et al., 2009; Hernaez et al., 2011; Ballestri et al., 2015); (2) there is no excessive alcohol consumption (defined as >140 g/week for male or 70 g/week for female, respectively) (Zeng et al., 2008); and (3) there are no competing etiologies for HS or chronic liver disease(CLD). Body mass index (BMI) was calculated as weight divided by height squared (kg/m^2^). All participants underwent plasma biochemistry, hemograms, and virological testing for HBV and HCV. Liver function tests [ALT, AST, γGT, TBA, total protein (TP), albumin(ALB), ALP, total bilirubin (TB), direct bilirubin (DB), and indirect bilirubin (IB)], fasting plasma glucose(FPG), low-density (LDL) lipoprotein, high-density lipoprotein (HDL), total cholesterol(TC), triglyceride(TG), and uric acid(UA) were measured using an ADVIA 2400 Clinical Chemistry System (Siemens, Germany). Total and direct bilirubin were measured by Vanadate oxidation method (Siemens, Germany). WBC, red blood cell (RBC), platelet (PLT), and hemoglobin (HB) were measured using XT-1800i Automated Hematology Analyzer (Sysmex, Japan). All biochemical tests were carried out in the same laboratory with standardized laboratory methods.

### DNA Extraction, Primer Design, and PCR Amplification

Genomic DNA was extracted from whole blood using a QIAamp DNA Blood Mini Kit (QIAGEN, Germany) according to the manufacturer's protocol. The *UGT1A1* gene promoter and first exon were PCR amplified and sequenced as previously described (Huang et al., 2002). Briefly, PCR conditions were as follows: 35 cycles at 94°C for 30 s, 60°C for 30 s, and 72°C for 30 s. The amplified products were purified from agarose gel and sequenced via an ABI3730XL sequencer (Applied Biosystems, Foster City, CA, USA).

### Statistical Analysis

Continuous variables were expressed as mean and standard deviation (SD) and then statistically analyzed using Student's *t*-test or one-way ANOVA. Categorical variables were analyzed using the chi-squared (χ2) test. Stepwise linear regression models were used to detect the effects of SNPs with total, indirect, and direct bilirubin levels. Candidate covariates in stepwise selection included the two *UGT1A1* variants (UGT1A1^*^6 and UGT1A1^*^28), sex, age, WBC, HB, PLT, ALB, TBA, and BMI. Binary logistic regressions were used to calculate ORs and the 95% confidence interval (CI) for NAFLD of bilirubin layering and *UGT1A1* variants after adjustment for the effects of sex, age, WBC, ALT, and BMI. All statistical analyses were performed using SPSS version 23.0 (SPSS, Chicago, IL, USA). A two-tailed *P*-value < 0.05 was considered statistically significant.

## Results

### Demographic Characteristics

Clinical characteristics stratified by *UGT1A1* genotypes are presented in Table 1. Among 403 enrolled individuals, the mean age was 37.3 ± 10.0 years old and 36.7% are men. Individuals in the study distributed into 4 genotype groups: 162 (40.2%) homozygous for wild-type genotypes, 87 (21.6%) with variant UGT1A1^*^6 genotypes, 126(31.3%) with variant UGT1A1^*^28 genotypes, and 28 (6.9%) with compound variant genotypes. The minor allele frequencies (MAF) of the *UGT1A1* variants were 15.1% (UGT1A1^*^28) and 21.5% (UGT1A1^*^6). UGT1A1^*^6 was in low degree of linkage disequilibrium with UGT1A1^*^28 (D′ = 1.000; *r*^2^ = 0.049). Except for BMI, there were no obvious difference among the four genotype groups in respect to ALT, AST, TP, ALB, TBA, ALP, γGT, WBC, HB, PLT, FPG, TC, TG, HDL, LDL, UA, and diastolic blood pressure (Table 1).

**Table 1:** Basic characteristics of the participants stratified by the *UGT1A1* Genotypes.

**Variables**	**Genotypes**				***P*****-value**		
	**Wild-type**	UGT1A1*6	UGT1A1*28	UGT1A1*6-*28	***P^**6/*wild***^***	***P^**28/*wild***^***	***P^***com*/*wild***^***
	**(*n* = 160; 40.2%)**	**(*n* = 87; 21.6%)**	**(*n* = 126; 31.3%)**	**(*n* = 28; 6.9%)**		
Sex, Female (%)	105 (64.8%)	56 (64.4%)	78 (61.9%)	16 (57.1%)	0.944	0.611	0.436
Age (years)	36.2 ± 9.1	38.9 ± 11.7	37.4 ± 9.7	38.1 ± 10.8	0.045	0.312	0.357
FPG (mmol/L)	4.75 ± 0.51	4.80 ± 0.45	4.87 ± 0.81	4.87 ± 0.64	0.527	0.093	0.339
TC (mmol/L)	4.29 ± 0.70	4.37 ± 0.82	4.37 ± 0.87	4.87 ± 0.64	0.490	0.398	0.479
TG (mmol/L)	1.27 ± 0.72	1.30 ± 0.86	1.47 ± 1.06	1.28 ± 0.80	0.770	0.060	0.956
HDL (mmol/L)	1.34 ± 0.30	1.34 ± 0.29	1.32 ± 0.30	1.31 ± 0.31	0.764	0.376	0.519
LDL (mmol/L)	2.51 ± 0.60	2.55 ± 0.79	2.56 ± 0.72	2.44 ± 0.54	0.666	0.539	0.673
UA (umol/L)	299.9 ± 74.0	289.0 ± 76.9	310.9 ± 86.3	313.2 ± 83.8	0.308	0.263	0.443
BMI (kg/m^2^)	22.7 ± 3.1	23.6 ± 3.6	24.2 ± 3.6	23.6 ± 3.1	0.055	< 0.001	0.196
Systolic (mmHg)	116.1 ± 16.8	118.5 ± 16.7	117.8 ± 16.9	122.8 ± 22.3	0.321	0.425	0.074
Diastolic (mmHg)	72.1 ± 11.0	73.7 ± 10.2	75.2 ± 11.4	73.4 ± 11.6	0.290	0.023	0.575
ALT ± SD (U/L)	17.6 ± 7.9	17.4 ± 7.0	19.0 ± 9.1	17.5 ± 6.7	0.804	0.144	0.929
AST ± SD (U/L)	17.7 ± 3.9	18.4 ± 4.6	18.2 ± 4.8	17.6 ± 3.9	0.218	0.292	0.888
TP ± SD (g/L)	75.9 ± 3.8	75.9 ± 4.4	75.9 ± 4.1	76.4 ± 4.3	0.869	0.975	0.557
Alb ± SD (g/L)	44.5 ± 2.3	44.6 ± 2.7	44.4 ± 2.7	44.8 ± 2.3	0.922	0.594	0.617
TBA ± SD (umol/L)	5.73 ± 2.41	5.91 ± 2.52	5.62 ± 2.37	5.43 ± 1.25	0.581	0.720	0.582
ALP ± SD (U/L)	65.9 ± 18.9	65.7 ± 15.1	68.0 ± 20.6	65.8 ± 15.6	0.928	0.338	0.986
γGT ± SD (U/L)	18.1 ± 10.2	17.9 ± 10.9	19.3 ± 11.6	18.5 ± 10.8	0.923	0.345	0.832
WBC ± SD(× 10^9^/L)	6.26 ± 1.34	6.22 ± 1.53	6.14 ± 1.28	5.88 ± 1.25	0.830	0.479	0.177
Hb ± SD (g/L)	135.2 ± 13.8	134.9 ± 14.1	136.1 ± 15.1	137.0 ± 16.0	0.923	0.581	0.529
PLT ± SD(× 10^9^/L)	227.1 ± 50.2	229.5 ± 47.4	235.7 ± 49.5	221.6 ± 53.8	0.713	0.145	0.591

Wild-type genotypes were homozygous, without either UGT1A1^*^6 or UGT1A1^*^28 variants. Variant UGT1A1^*^6 includes carriage of heterozygous or homozygous UGT1A1^*^6 variant allele. Variant UGT1A1^*^28 genotypes included heterozygous or homozygous UGT1A1^*^28 variants. The UGT1A1^*^6-^*^28 compound variant genotypes carry both UGT1A1^*^6 and UGT1A1^*^28 variants, either heterozygous or homozygous. P-values were calculated between groups by using analysis of variance for continuous variables and the χ^2^ test for categorical variables.

### The Effects of the *UGT1A1* Variants on Bilirubin Levels

We examined the effects of the variants (UGT1A1^*^28 and UGT1A1^*^6) on total, direct, and indirect bilirubin levels (Table 2). Total bilirubin levels were correlated with carriage of 1 and 2 copies of the variant alleles: compound heterozygotes (22.1 ± 12.6 umol/L), UGT1A1^*^6 (17.8 ± 8.6 umol/L), or UGT1A1^*^28 (16.4 ± 7.4 umol/L), and wild-type genotype (13.0 ± 4.9 umol/L), *P* < 0.001. The correlation between carriages of variant *UGT1A1* was similar for the indirect and direct bilirubin groups (*P* < 0.001, Table 2). The overall incidence of NAFLD was 18.9%, consisted with the reported incidence in China (Fan et al., 2017), while the distribution of incidence of NAFLD was 21.4, 17.2, 25.4, and 14.2% for compound heterozygotes, UGT1A1^*^6 carriers, UGT1A1^*^28 carriers, and non-carriers, respectively. There was a significant difference in incidence of NAFLD between the non-carrier group and UGT1A1^*^28 carrier group (*P* = 0.016, Table 2).

**Table 2:** The *UGT1A1* genotypes and bilirubin levels.

**Variables**	**Genotypes**				***P*****-value**		
	**Wild-type**	UGT1A1*6	UGT1A1*28	UGT1A1*6-*28	***P^**6/*wild***^***	***P^**28/*wild***^***	***P^***com*/*wild***^***
	**(*n* = 160; 40.2%)**	**(*n* = 87; 21.6%)**	**(*n* = 126; 31.3%)**	**(*n* = 28; 6.9%)**		
TB ± SD (μmol/L)	13.0 ± 4.9	17.8 ± 8.6	16.4 ± 7.4	22.1 ± 12.6	< 0.001	< 0.001	< 0.001
IB ± SD (μmol/L)	8.1 ± 3.3	11.6 ± 6.2	10.3 ± 4.2	14.3 ± 10.4	< 0.001	< 0.001	< 0.001
DB ± SD (μmol/L)	4.9 ± 1.7	6.2 ± 2.7	6.1 ± 3	7.7 ± 2.8	0.001	0.002	< 0.001
NAFLD (%)	14.2%	17.2%	25.4%	21.4%	0.524	0.016	0.485

The UGT1A1^*^6-^*^28 compound variant genotypes carry both UGT1A1^*^6 and UGT1A1^*^28 variants, either heterozygous or homozygous. P-values were calculated between groups by using ANOVA for continuous variables and the χ^2^ test for categorical variables.

*UGT1A1* variant genotypes explained a substantial portion of the variation in bilirubin levels (Table 3). Using a stepwise regression model selection procedure, bilirubin levels were found to be influenced by the *UGT1A1* variants as well as age, hemoglobin levels, platelet count, white blood count albumin, and total bile acid. With these co-factors being adjusted, UGT1A1^*^28 explained 5.6% and UGT1A1^*^6 explained 7.1% of the variation of total bilirubin levels. Similar results were observed for correlation with IB and DB (Table 3). In summary, the two common variants combined explain 12.7% of TB, 11.4% of IB, and 10.2% of DB levels.

**Table 3:** Genetic, demographic and biochemical factors that affect bilirubin levels.

**Explanatory variables**	**Total bilirubin(TB)**			**Indirect bilirubin(IB)**			**Direct bilirubin (DB)**		
	**Coefficient**	***R*^**2**^**	***P*-value**	**Coefficient**	***R*^**2**^**	***P*-value**	**Coefficient**	***R*^**2**^**	***P*-value**
UGT1A1*28	3.743 ± 0.577	5.6%	3.5%10^−10^	2.469 ± 0.383	4.8%	4.5%10^−10^	1.269 ± 0.212	4.2%	6.3%10^−9^
UGT1A1*6	2.908 ± 0.476	7.1%	2.9%10^−9^	1.869 ± 0.316	6.6%	8.8%10^−9^	1.032 ± 0.175	6.0%	9.7%10^−9^
Sex(Male)	1.599 ± 0.959	0.6%	0.096	1.320 ± 0.637	1.1%	0.039	0.277 ± 0353	0.1%	0.433
Age (years)	0.136 ± 0.032	2.9%	2.2%10^−5^	0.115 ± 0.021	10.0%	8.7%10^−8^	0.021 ± 0.012	0.6%	0.072
WBC (%10^9^/L)	−0.388 ± 0.215	3.1%	0.072	−0.183 ± 0.143	0.3%	0.201	−0.204 ± 0.079	5.8%	0.010
HB (g/L)	0.150 ± 0.033	9.4%	8.0%10^−6^	0.101 ± 0.022	6.3%	6.0%10^−6^	0.049 ± 0.012	8.9%	5.7%10^−5^
PLT (%10^9^/L)	−0.018 ± 0.006	1.8%	0.002	−0.010 ± 0.004	1.9%	0.008	−0.008 ± 0.002	2.8%	3.7%10^−5^
ALB (g/L)	0.512 ± 0.127	1.6%	6.8%10^−5^	0.306 ± 0.088	2.6%	3.2%10^−4^	0.206 ± 0.047	3.7%	1.4%10^−5^
TBA (umol/L)	−0.391 ± 0.115	4.8%	0.001	−0.282 ± 0.077	4.2%	2.7 × 10^−4^	−0.108 ± 0.042	1.8%	0.011
BMI (kg/m^2^)	−0.144 ± 0.089	0.7%	0.104	−0.069 ± 0.059	0.3%	0.245	−0.076 ± 0.033	1.0%	0.021

### Associated of Bilirubin Level With the Risk of NAFLD

We then analyzed the plasma bilirubin levels (TB, IB, and DB) and the distribution of *UGT1A1* genotypes with or without NAFLD (Table 4). Of 403 individuals, 76 (19%) were diagnosed as NAFLD, close to the 25% population prevalence of NAFLD in Asia (Fan et al., 2017). However, there was no significant difference in TB, IB, and DB levels between the NAFLD and non-NAFLD groups, either in the crude or fully adjusted model (*P* > 0.30, Table 4). Furthermore, there were no significant difference in the incidence of NAFLD among the 4 quartile groups with different levels of TB, IB, and DB, which further support no association between plasma bilirubin level (TB, DB, and IB) and NAFLD (*P* > 0.15, Supplementary Table 1).

**Table 4:** Distribution and multivariate analysis of *UGT1A1* genotypes for participants with and without NAFLD.

**Explanatory variables**	**NAFLD**	**Non-NAFLD**	***P*-value**	**OR (95%CI)**	***P*-value**	**OR (95% CI)_**adjusted**_**	***P-value_***adjusted***_***
	**(*n* = 76)**	**(*n* = 327)**					
TB (μmol/L)	16.2 ± 8.6	15.6 ± 7.5	0.548	1.009 (0.979–1.041)	0.547	1.028 (0.971–1.088)	0.349
DB (μmol/L)	5.9 ± 4.7	5.7 ± 2.4	0.622	1.025(0.949–1.107)	0.526	1.028 (0.861–1.227)	0.763
IB (μmol/L)	10.2 ± 4.9	9.9 ± 5.5	0.523	1.011(0.968–1.057)	0.622	1.045 (0.962–1.234)	0.300
**VARIANT** UGT1A1*6							
G/G	38 (50.0%)	211 (64.5%)	1.000				
G/A or A/A	38 (50.0%)	116 (35.5%)	0.019	1.819 (1.099–3.009)	0.020	1.638 (0.780–3.442)	0.193
**VARIANT** UGT1A1*28							
6/6	55 (72.4%)	233 (71.3%)	1.000				
6/7 or 7/7	21 (27.6%)	94 (28.7%)	0.846	0.946 (0.542–1.652)	0.846	1.395 (0.614–3.170)	0.427
**COMPOUND VARIANT GENOTYPES**							
6/6; G/G or G/A; 6/6 or 6/7; G/G	70 (92.1%)	305 (93.3%)	1.000				
G/A or A/A and 6/7 or 7/7	6 (7.9%)	22 (6.7%)	0.719	1.118 (0.465–3.040)	0.719	2.131 (0.589–7.704)	0.249

*Systolic blood pressure, diastolic blood pressure, fasting plasma glucose, total cholesterol, triglyceride, low-density lipoprotein, high-density lipoprotein, and uric acid were also included in the regression model and were not significant (each P > 0.05). Adjusted for age, sex, ALT, WBC, and BMI*.

### The Association Between the *UGT1A1* Variants and NAFLD

We used Mendelian randomization to determine if *UGT1A1* variant alleles differentially distribute between NAFLD cases and Non-NAFLD controls. The frequency of UGT1A1^*^6 variant was significantly higher in NAFLD group [50.0%; (OR = 1.82; 95% CI, 1.09–3.01; *P* = 0.02)] than in the non-NAFLD (35.5%), when compare with GG genotype (wild-type). However, in fully adjusted multivariate model, the association was attenuated and no longer significant (OR = 1.64, 95%CI, 0.78–3.44; *P* = 0.19) (Table 4). The prevalence of UGT1A1^*^28 genotypes or compound heterozygous genotypes did not differ significantly between the NAFLD and non-NAFLD groups in the crude analysis and in the fully adjusted model.

## Discussion

In the present study, we assessed the effects of *UGT1A1* variants and bilirubin levels on NAFLD. Two common variants (UGT1A1^*^6 and UGT1A1^*^28) were significantly associated with increased plasma bilirubin levels (TB, IB, and DB). However, these genetic variants and genetically elevated bilirubin levels (TB, IB, and DB) were not associated with decreased risk of NAFLD. Based on a Mendelian randomization approach, our data suggest that increased bilirubin levels is unlikely a causal factor for a reduced risk of NAFLD.

In our study, the frequencies of UGT1A1^*^28 and UGT1A1^*^6 variant alleles were 15.1 and 21.5%, respectively, similar to previously reported MAF in Asian populations (Huang et al., 2004; Memon et al., 2016). Previous studies (Bosma et al., 1995; Teng et al., 2007) have shown that UGT1A1^*^28 and UGT1A1^*^6 decrease the expression levels and activity of *UGT1A1*, thereby increasing TB levels. In this study, we found that these variants not only raise TB levels but also increase DB and IB levels. This is in agreement with a previous finding that UGT1A1^*^6 variant can increase urobilinogen levels, which correlates with DB levels (Kataoka et al., 2011). We found that the effect of UGT1A1^*^6 explains 7.1% of the total variation of TB levels, slightly higher than the previous results from a case-control study (5.2%) (Lin R. et al., 2009) and a GWAS (4.5%) (Dai et al., 2013) in Chinese. Further, we found that UGT1A1^*^28 had stronger effect on bilirubin levels (TB, IB, and DB) than either rs6742078 or rs887829 reported in the GWAS study (Chen et al., 2012; Dai et al., 2013). As rs6742078 and rs887829 are in high linkage disequilibrium with UGT1A1^*^28, it is likely that the reported GWAS signal for rs6742078 or rs887829 is tracking the functional UGT1A1^*^28 variant (Johnson et al., 2009; Yang et al., 2015). Moreover, the UGT1A1^*^28 and UGT1A1^*^6 (rs4148323) explain 12.7% of the total variation of TB levels, and *UGT1A1* is the only major gene that control total bilirubin variance in different populations (Johnson et al., 2009; Dai et al., 2013). The proportion of total phenotypic variance in most studied traits explained by the collective effects of known, common variants (*R*^2^) is rarely >10% but often >1%(Pierce et al., 2011), e.g., C-reactive protein, urate, lipids, triglycerides and FPG, as reviewed by Pierce et al. (2011). Therefore, the two variables are appropriate instruments for Mendelian Randomization analysis.

At present, there are two unresolved questions regarding the relationship between bilirubin levels and NAFLD. First, is there a relationship between the bilirubin levels and NAFLD? Two studies (Hjelkrem et al., 2012; Salomone et al., 2013) based on liver pathological diagnosis reported no significant association of bilirubin levels with NAFLD without non-alcoholic steatohepatitis (NASH), whereas a significantly lower prevalence of unconjugated hyperbilirubinemia in patients with histopathological evidence of NASH. Besides, there is a protective effect of elevated bilirubin level was found in three studies on risk of liver ultrasonography-diagnosed NAFLD including Han Chinese retirees (Tian et al., 2016), middle aged Korean Workers (Chang et al., 2012), and people for routine health checkups in Korean (Kwak et al., 2012). Whether the study on bilirubin and NAFLD were negative or positive, it has important value for clinical, and scientific research.

Furthermore, Variant UGT1A1^*^6 genotypes were associated with a lower risk of NAFLD in obese children, while the variant UGT1A1^*^28 genotypes and total bilirubin level were not significantly associated with the occurrence of pediatric NAFLD (Lin Y. C. et al., 2009). In our study, the *UGT1A1* variants (UGT1A1^*^6 and UGT1A1^*^28) and bilirubin level were not significantly associated with the risk of NAFLD in Han Chinese adult in Mendelian randomization trial. The inconsistent results could be owing to differences in study designs, genetic background, age, sex, severity of the disease and other variables. Second, which type of bilirubin is related to lower risk of NAFLD? Total bilirubin (IB and DB were not measured) was significantly associated with the incidence of NAFLD in some studies (Kwak et al., 2012; Puri et al., 2013), while direct bilirubin was associated with reduced NAFLD risk in other two studies (Chang et al., 2012; Tian et al., 2016). We did not observe an association between genetically bilirubin level (TB, IB, and DB) and risk of NAFLD. Of course, further studies are certainly needed to address the above issues.

Our study meets the three assumptions for Mendelian randomization method (Figure 1) (Johansen and Hegele, 2013; Emdin et al., 2017): (1) the genetic variant is related with the risk factor (bilirubin). In our study, the variants of UGT1A1^*^28 and UGT1A1^*^6 were strongly associated with increased TB, DB, and IB levels (each *P* < 0.001); (2) the genetic variant is not related with confounders. In our study, except for the bilirubin levels, other clinical variables were no significant different among the four *UGT1A1* genotype groups; (3) the genetic variant affect the consequence (NAFLD) only through the risk factor (bilirubin). *UGT1A1* is the only enzyme catalyzing the generation of water-soluble bilirubin glucuronides in hepatocytes (Memon et al., 2016; Wagner et al., 2018). UGT1A1^*^6 and UGT1A1^*^28 were also not in linkage disequilibrium with the known genes [e.g., *PNPLA3 and TM6SF2* (Macaluso et al., 2015; Fan et al., 2017)] that predispose to NAFLD. Moreover, in order to enhance statistical power of Mendelian randomization (Emdin et al., 2017), two variants in *UGT1A1* gene (UGT1A1^*^6 and UGT1A1^*^28) that affect the bilirubin levels the most were included in the analysis. To our knowledge, this is the first Mendelian randomization trial to explore the association of elevated bilirubin levels and NAFLD. Our findings that lifelong elevated bilirubin levels as well as the bilirubin level-controlling genetic variants, which are unconfounded by socio-economic and/or environmental factors, did not support a role of bilirubin in protecting the occurrence of NAFLD.

This study has several limitations. First, the study is a single-center, small-sample study and the results should be validated in large-scale prospective studies; Second, this study only analyzed the two common variants in *UGT1A1* gene, and other SNPs related to bilirubin levels were not included in the study, such as rs2417940 in *SLCO1B3* gene, which reportedly explains only 0.64% of TB variation (Kang et al., 2010). Nevertheless, the two common variants combined (UGT1A1^*^6 and UGT1A1^*^28) explains 12.7% of TB variation and are the major genetic factors controlling bilirubin levels. Third, our study enrolled mainly mid-aged adults that may have lower incidence of NAFLD than elders, hence the lower statistical power. Nevertheless, the incidence of NAFLD in the study is consistent with the prevalence in China (~20%) (Fan et al., 2017) and the common *UGT1A1* variants tested strongly predict bilirubin levels as expected. Fourth, the UGT1A1^*^28 group had higher BMI and incidence of NAFLD than the wild-type group (Tables 1, 2), consistent with the finding that obesity increases incidence of NAFLD (Chang et al., 2016; Targher and Byrne, 2016). We thus adjusted BMI as a covariable for all association tests. We also found that BMI had none or minor effect on bilirubin levels (Table 3), unsupported of an *UGT1A1* variants-BMI-bilirubin-NAFLD pathway, a potential confounder for Mendelian randomization trial. Lastly, it is well-known that liver biopsy is the gold standard for diagnosis of NAFLD (Chalasani and Younossi, 2018). The diagnosis of NAFLD was based on liver ultrasonography in this study, which may lead to potential attenuation of the associations between the NAFLD and bilirubin levels due to its insensitivity in detection of mild fatty liver. Whereas, the B-type ultrasonography is reliable and accurate in detection of moderate-severe fatty liver with sensitivity of 85% and specificity of 94% (Hernaez et al., 2011), and has been a cost-effective tool for screening NAFLD in epidemiological studies (Chang et al., 2012; Tian et al., 2016) and clinical practice (Chalasani and Younossi, 2018). Of course, future well-powered studies using biopsy-proven NAFLDs will provide more definitive conclusion.

In conclusion, genetically elevated plasma bilirubin levels were not associated with reduced risk of NAFLD in Mendelian randomization trial of a general adult population. These data suggest that bilirubin is unlikely causally related with the risk of NAFLD.

## Author Contributions

LL, PA, and ZD designed this study. LL carried out the experiments and analyses with the help of XJ, XY, SZ, SL, YC, WA, and ZD. LL, PA, CW, and ZD drafted the manuscript. All others contributed to the revision of the manuscript. All the authors read and approved the final version of the manuscript.

### Conflict of Interest Statement

The authors declare that the research was conducted in the absence of any commercial or financial relationships that could be construed as a potential conflict of interest.

## Abbreviations

ALB: albumin
ALP: alkaline phosphatase
ALT: aminotransferase
AST: aspartate aminotransferase
BMI: Body mass index
DB: direct bilirubin
FPG: fasting plasma glucose
HB: hemoglobin
HDL: high-density lipoprotein
IB: indirect bilirubin
LDL: low-density lipoprotein
PLT: platelet
NAFLD: non-alcoholic fatty liver disease
NASH: non-alcoholic steatohepatitis
γGT: glutamyltransferase
TB: total bilirubin
TC: total cholesterol
TG: triglyceride
TBA: total bile acid
TP: total protein
UA: uric acid
*UGT1A1*: uridine diphosphate glucuronosyltransferase 1A1
WBC: white blood count.
