# Supplementary material for: Genetically Regulated Bilirubin and Risk of Non-alcoholic Fatty Liver Disease: A Mendelian Randomization Study
Source: Front Genet. 2018 Dec 18;9:662. doi: 10.3389/fgene.2018.00662 (PMC6305545; doi:10.3389/fgene.2018.00662)
Supplement: Supplementary file 1 [file Table_1.docx]

**Supplementary Table 1. Odds ratios for NAFLD occurrence by serum bilirubin levels quartiles**

|  | No. Sample (%) | No. NAFLD (%) | OR(95%CI)_crude_ | *P* value _crude_ | OR(95%CI) _adjusted_ | *P* value _adjusted_ | |  |
| --- | --- | --- | --- | --- | --- | --- | --- | --- |
| Total bilirubin (umol/L) | | | | | | | | |
| Q1 | 100(24.8%) | 19(19.0%) | 1.000 |  | 1.000 |  | |  |
| Q2 | 99 (24.6%) | 19(19.2%) | 1.012(0.499-2.053) | 0.973 | 1.213(0.396-3.708) | | 0.735 | |
| Q3 | 102(25.3%) | 18(17.6%) | 0.914(0.448-1.864) | 0.804 | 0.839(0.290-2.430) | | 0.746 | |
| Q4 | 102(25.3%) | 20(19.6%) | 1.040(0.517-2.092) | 0.913 | 1.358(0.439-4.197) | | 0.595 | |
| Indirect bilirubin (umol/L) | | | | | | | | |
| Q1 | 102(25.3%) | 18(17.6%) | 1.000 |  | 1.000 | |  | |
| Q2 | 102(25.3%) | 18(17.6%) | 1.000(0.487-2.054) | 1.000 | 1.147(0.374-3.516) | | 0.811 | |
| Q3 | 99(24.6%) | 18(18.2%) | 1.037(0.504-2.133) | 0.921 | 0.886(0.303-2.591) | | 0.825 | |
| Q4 | 100(24.8%) | 22(22.0%) | 1.316(0.657-2.637) | 0.438 | 1.587(0.530-4.753) | | 0.409 | |
| Direct bilirubin (umol/L) | | | | | | | | |
| Q1 | 103(25.6%) | 21(20.4%) | 1.000 |  | 1.000 | |  | |
| Q2 | 102(25.3%) | 21(20.6%) | 1.012(0.514-1.995) | 0.972 | 0.701(0.244-2.014) | | 0.510 | |
| Q3  Q4 | 99(24.6%)  99(14.6%) | 16(16.2%)  18(18.2%) | 0.753(0.367-1.544)  0.868(0.431-1.748) | 0.438  0.691 | 0.452(0.150-1.361)  0.975(0.324-2.930) | | 0.158  0.963 | |

Notes: The cutoff values of bilirubin quartiles total bilirubin (Q1:≤10.49, Q2:10.50-13.99, Q3:14.00-18.99, and

Q4:≥19.00umol/L), indirect bilirubin (Q1:≤6.39, Q2:6.40-8.89, Q3:8.90-12.49, and Q4:≥12.50umol/L) and direct

bilirubin (Q1:≤4.00, Q2:4.01-5.99, Q3:6.00-6.99, and Q4:≥7.00umol/L), respectively.

Adjusted for sex, ALT, WBC and BMI.
